# Supramolecular Diversity in Bis(acylhydrazone) Crystals: Linker Effects, Polymorphism, and Gelator Assemblies

**DOI:** 10.1021/acs.cgd.5c01576

**Published:** 2025-12-23

**Authors:** Justin J. Zhao, Natalie E. Pridmore, Toby J. Blundell, Alice C. Taylor, David K. Smith, Niccoló Cosottini, Martin A. Screen, Amy V. Hall

**Affiliations:** † Department of Chemistry, 3057 Durham University, Durham DH1 3LE, U.K.; ‡ Department of Chemistry, 8748 University of York, Heslington, York YO10 5DD, U.K.

## Abstract

A series of previously unknown bis­(acylhydrazone)­s with aliphatic (zero to four CH_2_ units) and aromatic (phenylene substituted) linkers was synthesized and structurally characterized. Aliphatic derivatives exhibited distinct conformational geometries and packing motifs, with linker length critically affecting hydrogen bond interactions and energies. Aromatic derivatives revealed three polymorphs of the *meta*-substituted structure with two of the forms related by temperature. Additionally, a bis­(acylhydrazone) low-molecular-weight gelator was crystallized, revealing a unique and impressive hydrogen-bonded framework with large water channels (952 Å^3^) and strong aliphatic and aromatic stacking interactions. These findings highlight the potential of bis­(acylhydrazone)­s in crystal engineering and supramolecular chemistry, especially in coformer design and selection, and supramolecular gelator applications.

## Graphical abstract

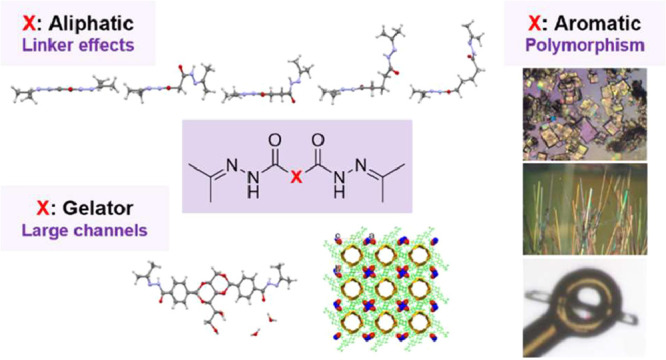

## Introduction

Noncovalent interactions are fundamental to the fields of crystal engineering and supramolecular chemistry. Hydrogen bonds, van der Waals forces, aromatic interactions, and halogen bonds are central to the formation of organic crystalline materials and their corresponding architectures, ranging from single-component molecular crystals,^1,2^ to cocrystals,^3,4^ to 3D self-assembled frameworks:^5,6^ each of which has the potential to exhibit polymorphism.^7^ Excellent candidates for cocrystallization and polymorphism studies are acylhydrazones (derived from the condensation of an acylhydrazide with an aldehyde or a ketone, Scheme 1), partly due to the potential of the hydrogen bond donor and acceptor functional groups in forming supramolecular synthons.^8^ In the Cambridge Structural Database (CSD, version 2025.2.0),^9^ searching for the acylhydrazone moiety in organic structures reveals almost 4500 hits. However, narrowing the CSD search to include terminal CH_3_ groups on the R_2_ and R_3_ groups revealed only 76 hits. Over half of the structures in the hitlist contain isonicotinic acid hydrazone derivatives, of which almost half are cocrystals, along with multiple cocrystal studies investigating the effect of the aliphatic coformer spacer length on crystal packing and properties.^10,11^ Additionally, acylhydrazone structures can display rare properties, with polar crystal polymorphs reported to undergo single-crystal-to-single-crystal transitions accompanied by violent crystal compressions and subsequent crystal shattering.^12^

**Scheme 1:**
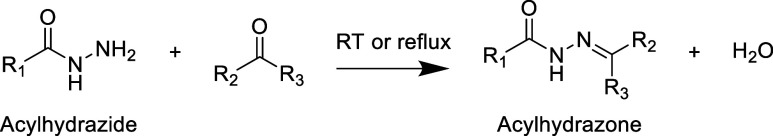
Acylhydrazides React with an Aldehyde or a Ketone to Give Acylhydrazones^a^

a Groups R_1_–R_3_ represent a hydrogen atom or any functional group.

Alongside acylhydrazones, bis­(acylhydrazone)­s are also apparent in materials science, typically with applications in sustainable and reversible polymers,^13^ but also as building blocks in inorganic complexes, acting as multidentate ligands.^14^ When expanding the CSD search to organic bis­(acylhydrazone)­s with terminal (CH_3_)_2_ groups in the CSD, there are only a handful of examples, highlighting the scarcity of these structures and the wealth of bis­(acylhydrazone) structures yet to be discovered, with the potential to aid in cocrystal formation, following the success of their mono derivatives. In this work, we have synthesized bis­(acylhydrazone)­s, termed DZ, with different length *n*-alkyl linker groups ranging from zero to four CH_2_ groups, and phenylene linker substitutions (*o* *rtho*, *meta*, *para*) (Figure 1). We have also explored the crystallization of a bis­(acylhydrazone) low-molecular-weight gelator (LMWG) based on 1,3:2,4-dibenzylidenesorbitol (DBS) (Figure 2).^15^ The acylhydrazide, DBS-CONHNH_2_, has been established as a highly effective hydrogelator,^16,17^ and recently, we reported its solid-state structure for the first time.^18^ Furthermore, it has previously been demonstrated that the resulting gels can easily be converted in situ into bis­(acylhydrazone)­s using dynamic covalent chemistry, by exposure to a variety of reactive aldehydes.^19^ As such, this LMWG is a platform for the formation of a diverse family of bis­(acylhydrazone)­s. Herein, we have characterized one of those bis­(acylhydrazone) derivatives and found vast water channels running throughout the structure, reminiscent of hydrogen-bonded organic frameworks (HOFs).

**Figure 1:**
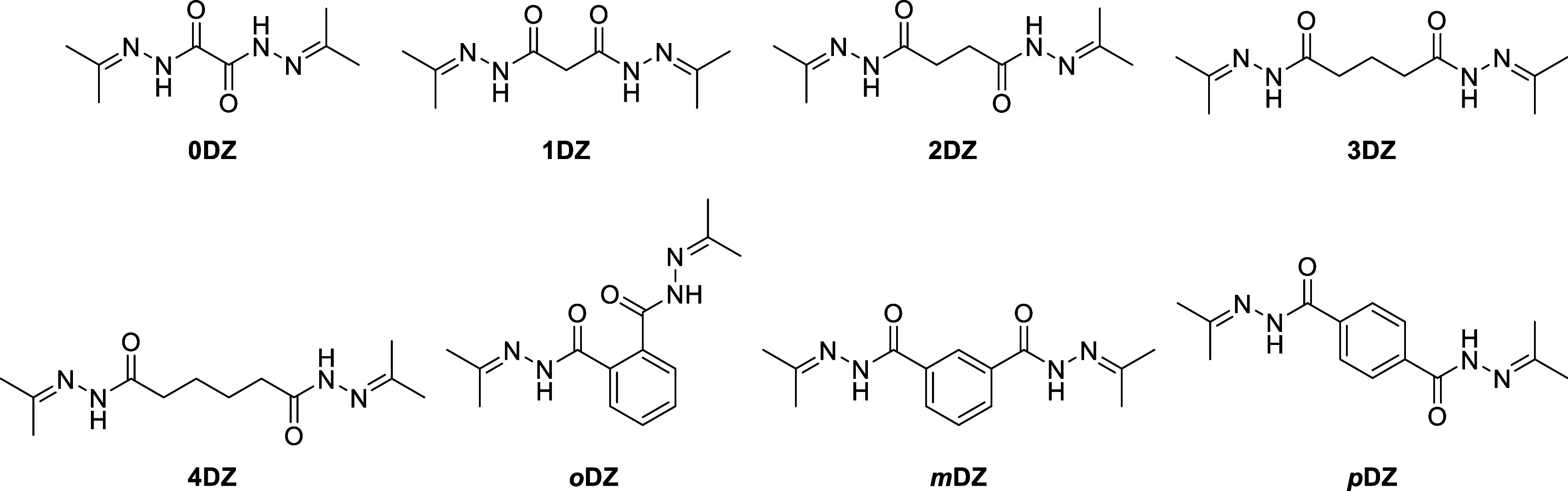
Bis­(acylhydrazone) structures included in this work, organized by linker length and type.

**Figure 2:**
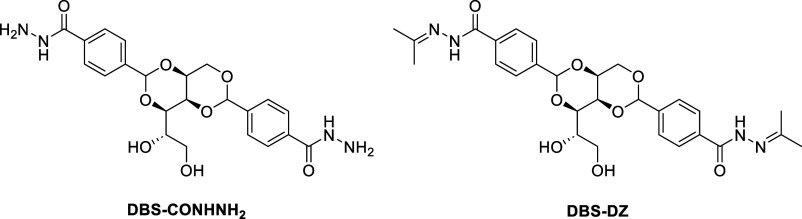
Structures of DBS-bis­(acylhydrazide) (DBS-CONHNH_2_) and DBS-bis­(acylhydrazone) (DBS-DZ).

## Results and Discussion

### Influence of *n*-Alkyl Linker Length on Molecular Conformation

Bis­(acylhydrazone)­s with terminal (CH_3_)_2_ groups and *n*-alkyl linker groups ranging from zero to four CH_2_ groups have been structurally characterized by X-ray diffraction (Table 1) and subsequently named 0DZ, 1DZ, 2DZ, 3DZ, and 4DZ. 0DZ has been reported in the CSD previously with refcode CUQPAF at a collection temperature of 295 K and a red crystal color.^20^ Conversely, in this work, clear colorless crystals of 0DZ were collected at 120 K, and though the unit cell parameters of the two structures differ slightly, the overall crystal packing of the two structures is very similar. 0DZ-3DZ crystallizes in a monoclinic crystal system (with space groups of either *P*2_1_/*n* or *P*2_1_/*c*), while 4DZ crystallizes as orthorhombic *Fdd*2. 4DZ also contains disorder over the planar CH_2_ linker groups, with both orientations present at 50% occupancies. An additional structure of 2DZ was collected and crystallized with four water molecules per 2DZ molecule (2DZ.4H_2_O). Further details of 2DZ.4H_2_O (and all crystal structures collected) can be found in the Experimental Section of the Supporting Information (SI).

**Table 1:** Crystallographic Parameters for 0DZ-4DZ^a^

	**0DZ**	**1DZ**	**2DZ**	**3DZ**	**4DZ**
emp. form.	C_8_H_14_N_4_O_2_	C_9_H_16_N_4_O_2_	C_10_H_18_N_4_O_2_	C_11_H_20_N_4_O_2_	C_12_H_22_N_4_O_2_
form. weight (g/mol)	198.23	212.26	226.28	240.31	254.33
habit	plate	needle	needle	needle	needle
crystal system	monoclinic	monoclinic	monoclinic	monoclinic	orthorhombic
space group	*P*2_1_/*n*	*P*2_1_/*c*	*P*2_1_/*n*	*P*2_1_/*n*	*Fdd*2
*a* (Å)	5.1745(5)	8.6380(4)	11.8912(7)	9.0318(5)	15.5691(9)
*b* (Å)	16.8291(15)	16.7078(7)	8.0793(5)	11.2712(6)	28.4277(16)
*c* (Å)	5.6742(5)	8.0144(3)	13.1797(7)	12.3742(7)	6.1100(4)
β (°)	95.851(4)	103.0230(10)	107.867(2)	91.673(2)	90
Z	2	4	4	4	8
*V* (Å^3^)	491.55(8)	1126.90(8)	1205.14(12)	1259.15(12)	2704.2(3)
*D*_c_ (g/cm^3^)	1.339	1.251	1.247	1.268	1.249
*R*_1_ (*I* > = 2σ(*I*))	0.0698	0.0532	0.0601	0.0541	0.1259

a All data were collected at 120 K.

The molecular conformations of the DZs are summarized in Figure 3. Structurally, 0DZ has a planar dihydrazone backbone (with an N1–C1–C2–N2 torsion angle of 180°) with out-of-plane methyl group arms at each end of the molecule. The difference of just one CH_2_ linker group results in a dramatically different molecular arrangement.^21,22^ The structure of 1DZ reveals *anti* and anticlinal conformations of the N1–C1–C2–C3 atoms (torsion angles of −166 and 96°) in the center of the molecule, allowing it to twist compared to the planar arrangement of 0DZ. For 2DZ, the N1–C1–C2–C3 and N2–C4–C3–C2 torsion angles reveal *anti* (175°) and gauche (84°) geometries, respectively, while the carbonyl carbon atoms and carbon linker groups (C1–C2–C3–C4) remain close to planar at −176°. In 3DZ, *anti* and anticlinal conformations of the N1–C1–C2–C3 atoms (torsion angles of −166 and 96°) are the same as in 1DZ; however, the conformations of the C1–C2–C3–C4 and C5–C4–C3–C2 are gauche, with torsion angles of 80 and 79°. The structure of 4DZ is comprised of planar CH_2_ linker groups (C2–C3–C4–C5 torsion angle of 179°), though when the carbonyl carbon atoms are included in the angle measurements, one side remains planar (C1–C2–C3–C4 torsion angle of 180°), while the other side (C6–C5–C4–C3) is gauche with a torsion angle of −69°. Both N1–C1–C2–C3 and N2–C6–C5–C4 conformations exist as anticlinal (with torsion angles of 142 and 125°). All aliphatic DZ structures have an *anti*-conformation over the hydrazone moiety (C–N–N1–C2), and therefore, it is the linker groups that determine the conformation and geometry of the bis­(acylhydrazone) molecule. Furthermore, there are no obvious differences between the conformations of DZ structures with odd or even numbers of CH_2_ linker groups in these specific structures.

**Figure 3:**
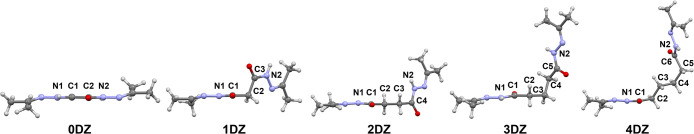
Molecular conformations of 0DZ-4DZ. The disorder of 4DZ over C2–C3–C4–C5 has been omitted for the sake of clarity.

Further to the aliphatic linker effects on the DZs, the two strongest interactions between DZ dimers were considered^23,24^ and subsequently characterized by their interaction type, followed by their graph-set analysis^25,26^ if hydrogen-bonded motifs were involved. These interactions are summarized in Figure 4. In the packing of 0DZ, aliphatic stacking interactions (primarily CH···C interactions with the terminal methyl groups between molecules) are the strongest (−41.9 kJ/mol), followed by *R*_2_^2^(10) hydrogen-bonded dimers (−39.1 kJ/mol) perpendicular to the aliphatic stacking direction. In 1DZ, the most favorable interaction is the *R*_1_^2^(5) dimers with an associated energy of −67.6 kJ/mol, followed by weaker *R*_2_^2^(8) hydrogen-bond dimers (−42.4 kJ/mol). The strongest interaction in 2DZ is the *R*_2_^2^(14) dimers with an associated energy of −86.3 kJ/mol, with the *R*_1_^2^(5) dimers following with an energy of −47.4 kJ/mol. The strongest interactions out of all the aliphatic DZs are the *R*_1_^2^(5) dimers in 3DZ, with a calculated energy of −92.3 kJ/mol, and these dimers twist the molecule throughout the 3D structure. The second most favorable interaction in 3DZ is the aliphatic stacking of the molecules (via weak CH···N and CH···C interactions) with an energy of −44.3 kJ/mol. Interestingly, for 4DZ, no ring hydrogen-bond dimers are included, only *C*_1_^1^(3) chains are at –45.8 and –45.6 kJ/mol. In summary, hydrogen-bonded dimers with ring motifs dominate in the aliphatic DZ structures, with the *R*_1_^2^(5) dimers showing the strongest interactions on average, particularly in 1DZ-3DZ, compared with the ring counterparts with two hydrogen bond donors and acceptors. Additional information such as calculation details, interaction descriptions, Hirshfeld surface analysis, and corresponding 2D fingerprint plots can be found in Table S1 and Figure S1.

**Figure 4:**
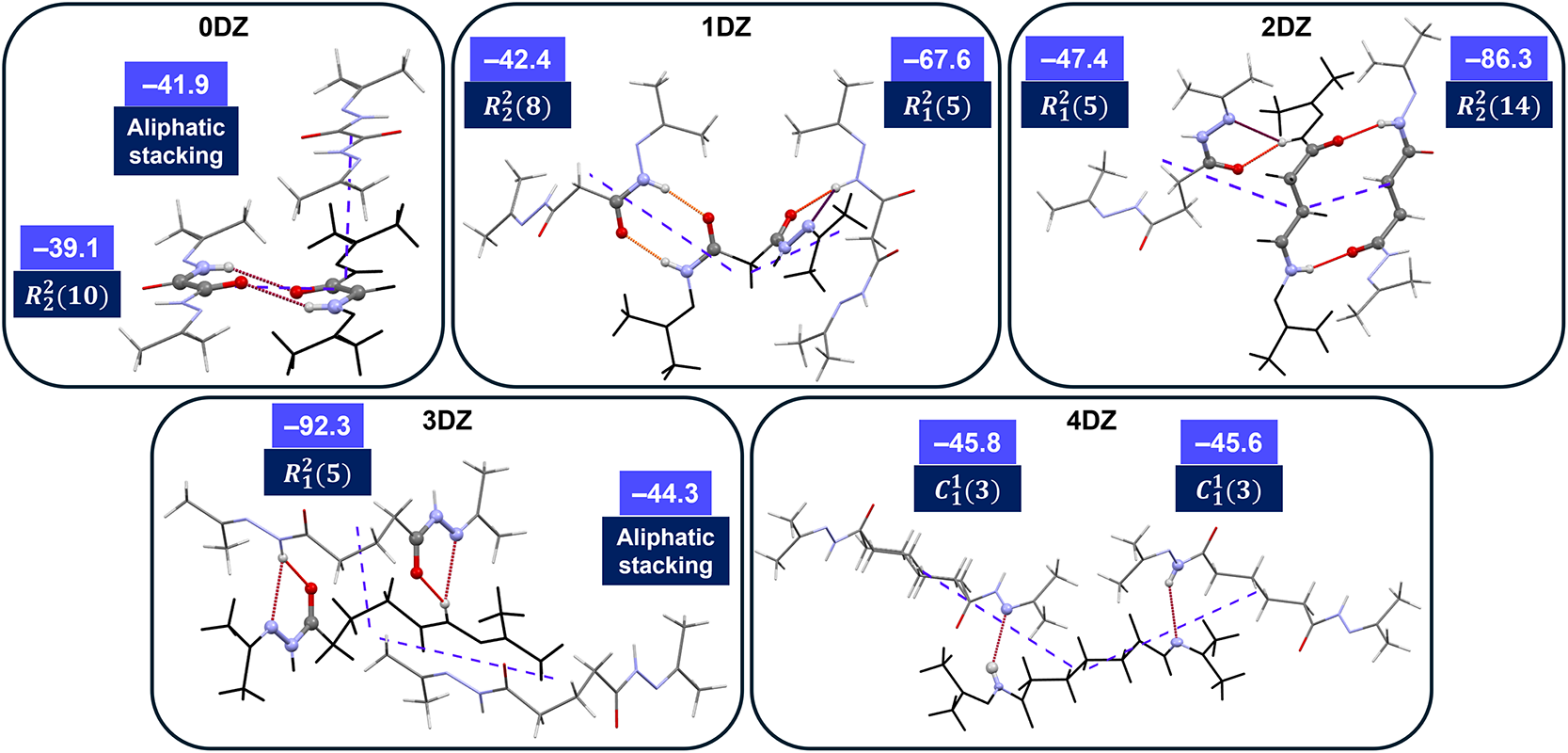
Two most favorable interactions (blue dashed lines) in each of the aliphatic DZ structures from the center molecule (in black). Hydrogen-bonding motifs and the atoms involved are highlighted in the ball and stick model, along with the corresponding graph set notation. All calculated energy values are in kJ/mol. Hydrogen bonds are colored on a scale corresponding to length: Short (yellow), mid (red), long (purple).

### Structural Diversity with Aromatic Linker Substitution

In addition to the aliphatic DZ structures, aromatic DZs were also considered, differing in having the hydrazone moiety in the *ortho* (*o*DZ), *meta* (*m*DZ), or *para* (*p*DZ) positions relative to the phenylene linker. For *oDZ*, only phthalhydrazide (IJOKIB)^27^ could be crystallized despite phthalhydrazide not being present in the starting material. Multiple crystallization experiments of 1,2-benzenedicarboxylic acid dihydrazide to obtain *oDZ* using different methods and solvents were attempted (and described in the SI); however, none yielded the desired product. Therefore, this section focuses only on the structures of *mDZ* and *pDZ*.

The cooling crystallization of *mDZ* resulted in large needles that transformed into small plates after 1 h. The X-ray structure of the plates was collected at 120 K; however, at the same temperature, the needles appear to undergo a phase transition and were subsequently collected at 200 K. Additionally, when the needles were submerged and remained in liquid nitrogen (77 K) and analyzed at the Diamond Light Source (DLS) at 100 K, a different, low-temperature structure was collected. Each structure of *mDZ* yields different packing polymorphs, with the two needle polymorphs related by temperature. Therefore, the stable plates collected at 120 K, the needles collected at 200 K, and the needles collected at 100 K are subsequently named Form I, Form II, and Form III, respectively (Table 2). The temperature-dependent needle polymorphs were recollected at the DLS and again confirm the Form II and III structures (additional details can be found in SI).

**Table 2:** Crystallographic Parameters for the *m*DZ Polymorphs and *p*DZ

	*m* **DZ**			*p* **DZ**
	**Form I**	**Form II**	**Form III**	
emp. form.	C_14_H_18_N_4_O_2_	C_14_H_18_N_4_O_2_	C_14_H_18_N_4_O_2_	C_14_H_18_N_4_O_2_
form. weight (g/mol)	274.32	274.32	274.32	274.32
temperature (K)	120.0	200.0	100.0	120.0
habit	square plate	needle	needle	plank
crystal system	triclinic	monoclinic	monoclinic	monoclinic
space group	*P-*1	*C*2/*c*	*P*2_1_ */c*	*P*2_1_/*c*
*a* (Å)	8.2836(4)	19.475(2)	8.4173(5)	11.3237(18)
*b* (Å)	8.3857(4)	8.9815(9)	8.1862(5)	7.8854(13)
*c* (Å)	20.6240(10)	8.2696(8)	20.0715(17)	7.9632(11)
α (°)	84.501(2)	90	90	90
β (°)	79.725(2)	100.066(3)	95.615(7)	107.616(5)
γ (°)	89.734(2)	90	90	90
Z	4	4	4	2
*V* (Å^3^)	1403.05(12)	1424.2(2)	1376.40(17)	667.71(18)
*D*_c_ (g/cm^3^)	1.299	1.279	1.324	1.344
*R*_1_ (*I* > = 2σ(*I*))	0.0442	0.0680	0.1167	0.0430

The structure of Form I contains two molecules per asymmetric unit that are ∼ 70 degrees to each other, each with alternating *R*_1_^2^(5) (two per dimer) and *R*_2_^2^(16) hydrogen-bonded dimers running throughout the structure. The *R*_1_^2^(5) hydrogen-bond dimers have a calculated energy of −117.4 kJ/mol compared to the *R*_2_^2^(16) dimers with a higher energy of −99.3 kJ/mol (similar energies exist for the other molecule of the asymmetric unit with *R*_1_^2^(5) and *R*_2_^2^(16) hydrogen-bonded dimers of −115.5 and −92.8 kJ/mol, respectively). Additional weak aromatic interactions occur within the dimers at centroid···centroid distances of 6.0 and 6.6 Å. For the needles, Form II crystallizes with half a molecule in the asymmetric unit and packs as continuous hydrogen-bonded ribbons consisting of two *R*_1_^2^(5) hydrogen bonds per dimer. The dimers contain moderate aromatic stacking with a centroid···centroid distance of 6.3 Å and an associated calculated energy of −109.6 kJ/mol. Similar to Form I, the Form III structure packs in continuous hydrogen-bonded ribbons, alternating between *R*_1_^2^(5) (two per dimer) and *R*_2_^2^(16) dimers, with calculated energies of −113.2 and −96.0 kJ/mol, respectively. Aromatic stacking is also observed in Form III, with centroid···centroid distances of 5.9 and 6.6 Å between rings of the hydrogen-bonded dimers.

Further to *m*DZ, plank crystals of *p*DZ crystallize in a monoclinic crystal system and were analyzed at 120 K. The packing of *p*DZ reveals *R*_1_^2^(5) dimers from one *p*DZ molecule going to two *p*DZ molecules orientated ∼30° to each other, resulting in a twisted hydrogen-bonded network. Each *R*_1_^2^(5) dimer is associated with a calculated energy of −61.7 kJ/mol, which is further reinforced by aromatic interactions, with a centroid···centroid distance of 5.6 Å, the shortest aromatic interaction of the aromatic DZs in this work. The crystal morphology and crystal packing of the *m*DZ polymorphs and *p*DZ are shown in Figure 5.

**Figure 5:**
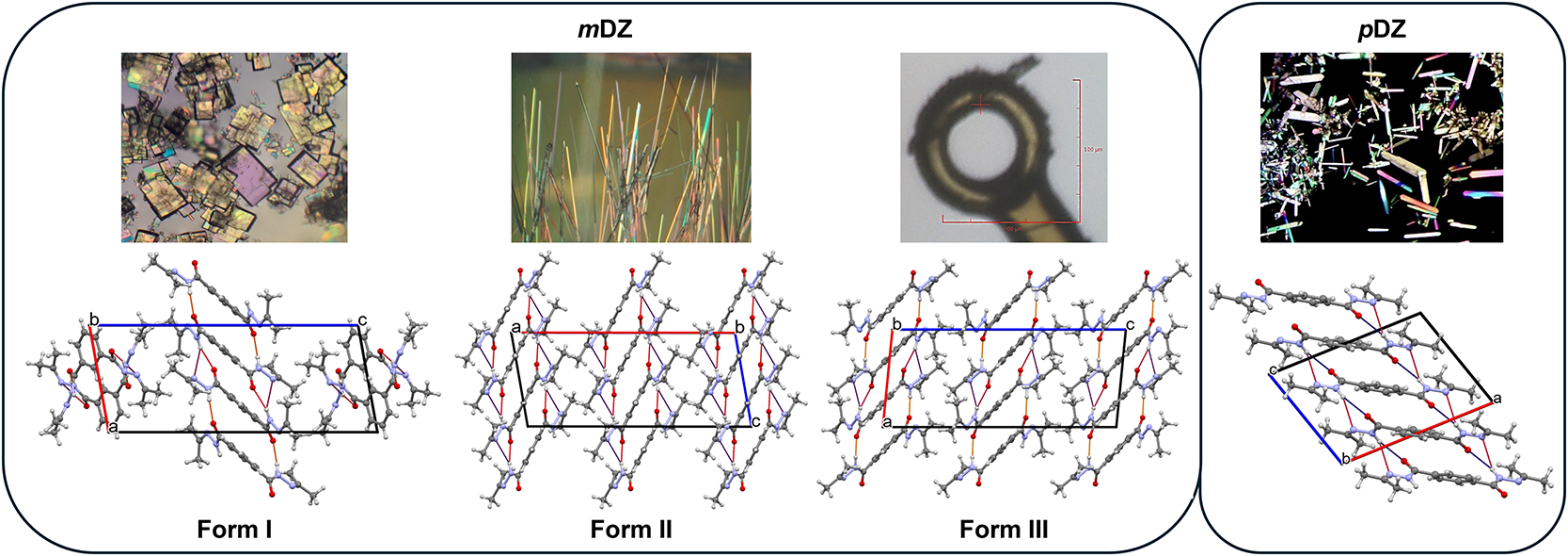
Crystal morphology and packing of the *m*DZ polymorphs and *p*DZ. Hydrogen bonds are colored on a scale corresponding to length: Short (yellow), mid (red), long (purple).

### Bis­(acylhydrazone) Supramolecular Gelator Assembly

Low-molecular-weight gelators in the field of supramolecular chemistry are often employed to crystallize metastable or hard-to-nucleate polymorphs of small molecules^28^ or as vehicles in drug formulation and delivery.^29^ Gels comprised of LMWGs with bis­(acylhydrazone) moieties exhibit wide-ranging applications, from catalysts,^30–32^ to metal sensing,^33–35^ and photoresponsive liquid crystals.^36^ However, solid-state structures of the LMWGs themselves are scarce due to their tendency to form 2D fibers in solution rather than crystallize. We recently disclosed the solid-state structure of a bis­(acylhydrazide) hydrogelator, DBS-CONHNH_2_.^18^ This LMWG is capable of reacting in the gel with a variety of carbonyl compounds to form self-assembling bis­(acylhydrazone)­s.^19^ Here, we reacted DBS-CONHNH_2_ powder with acetone at room temperature to yield small needle crystals of the bis­(acylhydrazone), named DBS-DZ (Figure 2). We reasoned that this might allow us to explore the assembly and packing of more structurally complex, self-assembling bis­(acylhydrazone)­s.

The X-ray structure of DBS-DZ revealed a water-rich framework. The asymmetric unit contains seven water molecules (two crystallographically modeled, five masked—see the SI for details) per DBS-DZ molecule (Figure 6A) in two channels, equating to 28 water molecules in the unit cell. The symmetry and packing of the structure reveal large channels, with one channel per unit cell containing crystallographically disordered water molecules, corresponding to a solvent-accessible volume of 952 Å^3^ (calculated in Olex2,^37^ using a spherical probe radius of 1.2 Å and a grid of approximately 0.2 Å) that act as one-dimensional water channels throughout the structure (Figure 6B). The modeled water molecules form separately defined, smaller water channels that adopt a helical hydrogen-bonded arrangement due to the *P*4_3_ symmetry present (Figure 6C). The modeled water molecules hydrogen bond to adjacent water molecules and to the carbonyl oxygen atom and nitrogen atom of one-half of the DBS-DZ molecule. Additional hydrogen bonds in the structure originate from the central sugar moiety of the gelator via OH···O and OH···N hydrogen bonds to neighboring DBS-DZ molecules. The aliphatic stacking of the sugar moieties and the aromatic stacking of the phenylene rings (centroid···centroid distances of 4.74 and 4.83 Å) are the most favorable interactions in the structure, with a calculated interaction of −148.9 kJ/mol, further reinforcing the favorable butterfly stacking of LMWG molecules, comparable to DBS itself.^38^

**Figure 6:**
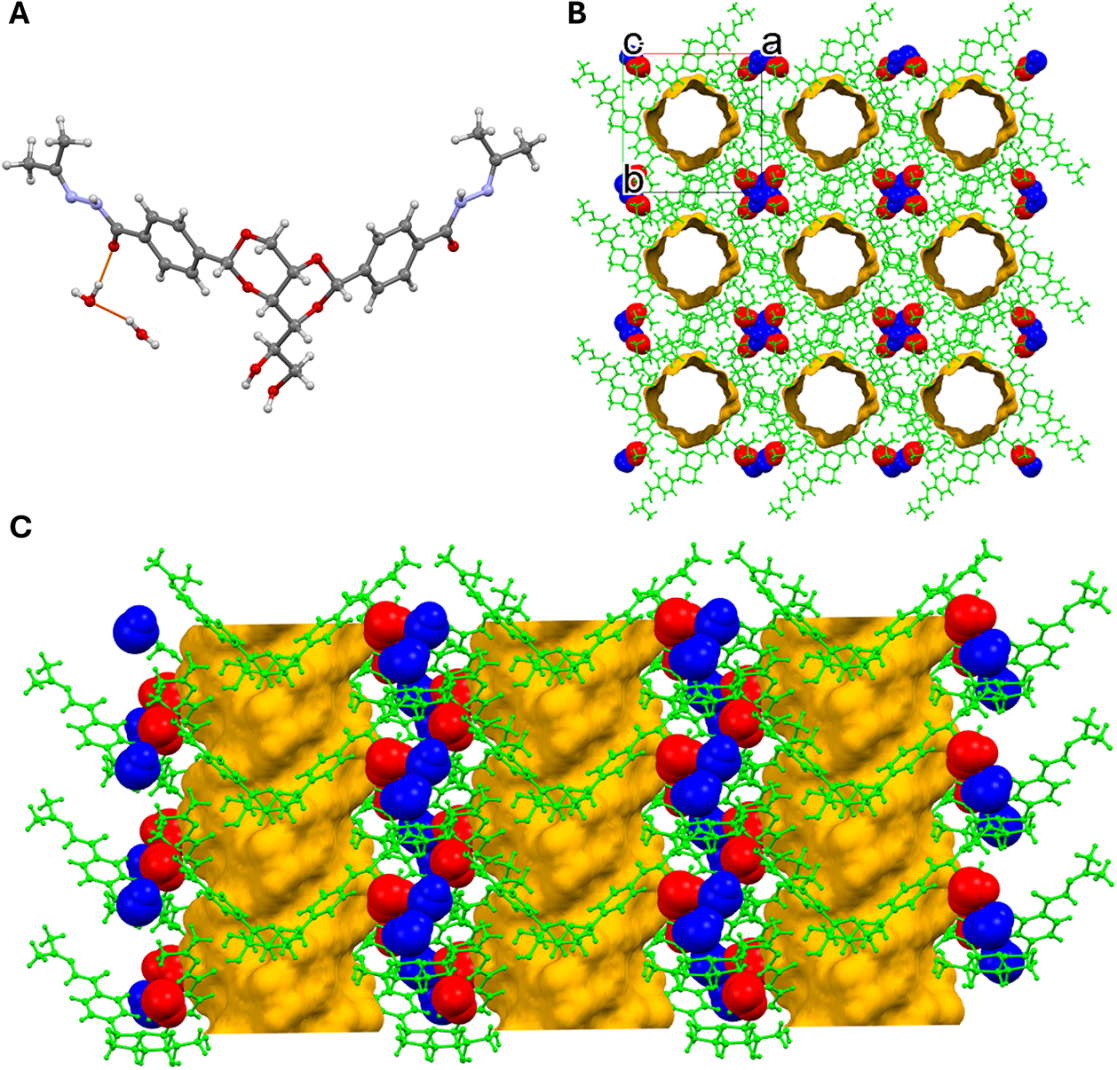
DBS-DZ: (A) The asymmetric unit with two modeled water molecules per DBS-DZ molecule; (B) the 3 × 3 × 3 crystal structure packing along the *c*-axis, exposing the large disordered water channels within the structure and the defined, smaller water channels found on the outside; (C) the crystal structure packing along the *b*-axis, displaying the helical nature of the gelator molecule and the hydrogen-bonded water molecules around the large channels. Structural elements in Figure 6B,C are colored by symmetry equivalence, and water molecules are displayed as spacefill style for clarity.

Due to the vast water content and hydrogen bonding capability of the LMWG, it is not surprising that the material is a hydrogelator. Out of 30 solvents tested, DBS-DZ only gels water at a gel concentration of 0.5 wt %/vol (information on the full solubility and gel screen of DBS-DZ can be found in the SI). The three-dimensional crystalline network upheld by noncovalent interactions and combined with large structural channels has been likened to HOFs,^6^ though no bis­(acylhydrazone) structures have been utilized in this way before; however, acylhydrazone moieties are extensively used in covalent organic frameworks^39^ and even used as linkers in metal organic frameworks.^40^ Therefore, possible applications for self-assembled bis­(acylhydrazone) gelator structures as ordered frameworks are extensive. Given the vast potential for structurally tuning the carbonyl component in the conversion of DBS-CONHNH_2_ into a bis­(acylhydrazone),^19^ this suggests that this approach may unlock an exciting new playground for the manipulation and assembly of ordered organic frameworks.

## Conclusion

This study demonstrates the structural diversity and supramolecular effects of bis­(acylhydrazone)­s driven by subtle variations in the *n*-alkyl linker length and aromatic substitution. The systematic exploration of aliphatic linkers (zero to four CH_2_ units) reveals distinct conformational preferences and packing motifs, with the linker influencing hydrogen bonding and stacking interactions. The discovery of multiple polymorphs of *m*DZ highlights the polymorphic potential of bis­(acylhydrazone)­s and their sensitivity to crystallization conditions. Most notably, the crystallization of a bis­(acylhydrazone) low-molecular-weight gelator, DBS-DZ, formed by the reaction of the LMWG DBS-CONHNH_2_ with acetone unveiled a water-rich framework with extensive hydrogen-bonding and aliphatic and aromatic stacking interactions. These findings not only expand the structural landscape of bis­(acylhydrazone)­s but also highlight their potential as functional building blocks in crystal engineering, coformer design and supramolecular materials. The highly possible tunability of bis­(acylhydrazone) derivatives formed by combining DBS-CONHNH_2_ with different carbonyl compounds suggests that this approach may underpin a versatile new class of ordered organic framework materials.

## Supplementary Material



## Notes

All crystal interactions and associated energy calculation details, Hirshfeld surface analyses, all experimental details and methodology for all structures in the manuscript, and crystallographic Information Files for all crystal structures discussed in the manuscript (PDF)

## Abbreviations

CSD: Cambridge Structural Database
DBS: 1,3:2,4-dibenzylidenesorbitol
DLS: Diamond Light Source
HOFs: hydrogen-bonded organic frameworks
LMWG: low-molecular-weight gelator
