# Supplementary material for: Supramolecular Diversity in Bis(acylhydrazone) Crystals: Linker Effects, Polymorphism, and Gelator Assemblies
Source: Cryst Growth Des. 2025 Dec 23;26(1):664–70. doi: 10.1021/acs.cgd.5c01576 (PMC12784328; doi:10.1021/acs.cgd.5c01576)
Supplement: Supplementary file 1 [file cg5c01576_si_001.pdf]

Supramolecular Diversity in Bis(acylhydrazone) Crystals: Linker Effects, Polymorphism, and Gelator Assemblies

Justin J. Zhao,[a] Natalie E. Pridmore,[a] Toby J. Blundell,[a] Alice C. Taylor,[a] David K. Smith,[b] Niccoló Cosottini,[b] Martin A. Screen,[a] Amy V. Hall[a]*

a. Department of Chemistry, Durham University, Durham, DH1 3LE, UK.

b. Department of Chemistry, University of York, Heslington, York, YO10 5DD, UK.

Interaction Energy Calculations

Interaction energy calculations for each experimental crystal structure were calculated using unified (UNI) pair-potential parameters, using the UNI Intermolecular Potentials[1, 2] functionality in Mercury (version 2025.3).[3] Using this method, potentials between a central molecule and the nearest 200 surrounding molecules are calculated, along with the total packing energy and convergence. Table S1 lists a breakdown of the stated interactions type for each DZ compound with the two lowest energies, along with their molecular center-center distance and corresponding graph set notation.

Compound	Mol. 1	Mol. 2	Centroid distance of Mol. 1 and 2 (Å)	Calculated energy (kJ/mol)	Intermolecular interaction type and description	Interaction distance (Å)	Graph set notation
0DZ	0	1	5.6742	–41.9477	Aliphatic stacking: $CH(CH_3)\cdots C$(carbonyl)	C···C 3.51	n/a
	0	2	5.1745	–39.1456	NH···O hydrogen-bonded dimer	N···O 3.20	$R_2^2(10)$
1DZ	0	1	4.1792	–67.6435	NH···O and NH···N hydrogen-bonded dimer	N···O 2.89; N···N 3.31	$R_1^2(5)$
	0	2	7.30659	–42.357	NH···O hydrogen-bonded dimer	N···O 2.87	$R_2^2(8)$
2DZ	0	1	4.02581	–86.2653	NH···O hydrogen-bonded dimer	N···O 3.02	$R_2^2(14)$
	0	2	5.89749	–47.4294	NH···O and NH···N hydrogen-bonded dimer	N···O 2.95; N···N 3.15	$R_1^2(5)$
3DZ	0	1	4.02574	–92.2625	NH···O and NH···N hydrogen-bonded dimer	N···O 3.01; N···N 3.16	$R_1^2(5)$
	0	2	6.99209	–44.3168	Aliphatic stacking: $CH(CH_3)\cdots N$(N=C) and $CH(CH_3)\cdots C$(N=C)	C···N 3.60; C···C 3.83	n/a
4DZ	0	1	8.25840	–45.8302	NH···N hydrogen-bonded chains	N···N 3.26	$C_1^1(3)$
	0	2	8.32773	–45.6268	NH···N hydrogen-bonded chains	N···N 3.26	$C_1^1(3)$
*m*DZ, Form I	0	1	4.03702	–117.397	NH···O and NH···N hydrogen-bonded dimer	N···O 3.11; N···N 3.27	$R_1^2(5)$

					Aromatic stacking	Ring centroid···Ring centroid 6.56	n/a
	2	3	4.07561	–115.509	NH···O and NH···N hydrogen-bonded dimer	N···O 3.16 and N···N 3.30	$R_1^2(5)$
					Aromatic stacking	Ring centroid···Ring centroid 5.96	n/a
	0	4	4.53558	–99.3368	NH···O hydrogen-bonded dimer	N···O 2.92	$R_2^2(16)$
	2	5	4.62658	–92.8291	NH···O hydrogen-bonded dimer	N···O 2.91	$R_2^2(16)$
*m*DZ, Form II	0	1	4.26572	–109.575	NH···O and NH···N hydrogen-bonded dimer	N···O 3.09; N···N 3.40	$R_1^2(5)$
					Aromatic stacking	Ring centroid···Ring centroid 6.3	n/a
	0	2	10.4732	–11.2715	Aliphatic stacking: $CH(CH_3)\cdots C(CH_3)$	C···C 3.90	n/a
*m*DZ, Form III	0	1	4.10789	–113.209	NH···O and NH···N hydrogen-bonded dimer	N···O 3.18; N···N 3.36	$R_1^2(5)$
					Aromatic stacking	Ring centroid···Ring centroid 6.62	n/a
	0	2	4.67302	–95.9691	NH···O hydrogen-bonded dimer	N···O 2.88	$R_2^2(16)$

					Aromatic stacking	Ring centroid···Ring centroid 5.86	n/a
*p*DZ	0	1	5.60339	–61.6595	NH···O and NH···N hydrogen-bonded dimer	N···O 3.53 and N···N 3.12	$R_1^2(5)$
					Aromatic interactions	Ring centroid···Ring centroid 5.60	n/a
	0	2	7.8854	–13.4156	Aliphatic stacking: CH(ring)···C(CH_3)	C···C 3.85	n/a
DBS-DZ	0	1	4.71885	–148.8770	Aliphatic stacking: CH(sugar)···O(sugar); CH(sugar)···C(ring); CH(ring)···C(carbonyl)	C···O 3.58; C···C 3.62-3.91; C···C 3.35-3.48	n/a
					Aromatic stacking	Ring centroid···Ring centroid 4.83 and 4.74	n/a
	0	2	15.3728	–59.4778	OH···O and OH···N hydrogen-bonded dimer	O···O 3.33; O···N 2.87	$R_1^2(5)$
					OH···O hydrogen bond	O···O 2.76	n/a
					Aliphatic stacking: CH(ring)··C(CH_3)	C···C 3.41	n/a

Table S1. A summary of the calculated intermolecular energies for the two strongest interactions per DZ molecule, from a central DZ molecule to the strongest nearby contact, along with their calculated distances, interaction types, and corresponding graph set notations.

Hirshfeld Surface Analysis

Hirshfeld surfaces (d_{norm}) and 2D fingerprint plots (d_e and d_i) for each crystal structure were calculated using CrystalExplorer (version 25.8)[4] and are shown in Figure S1.

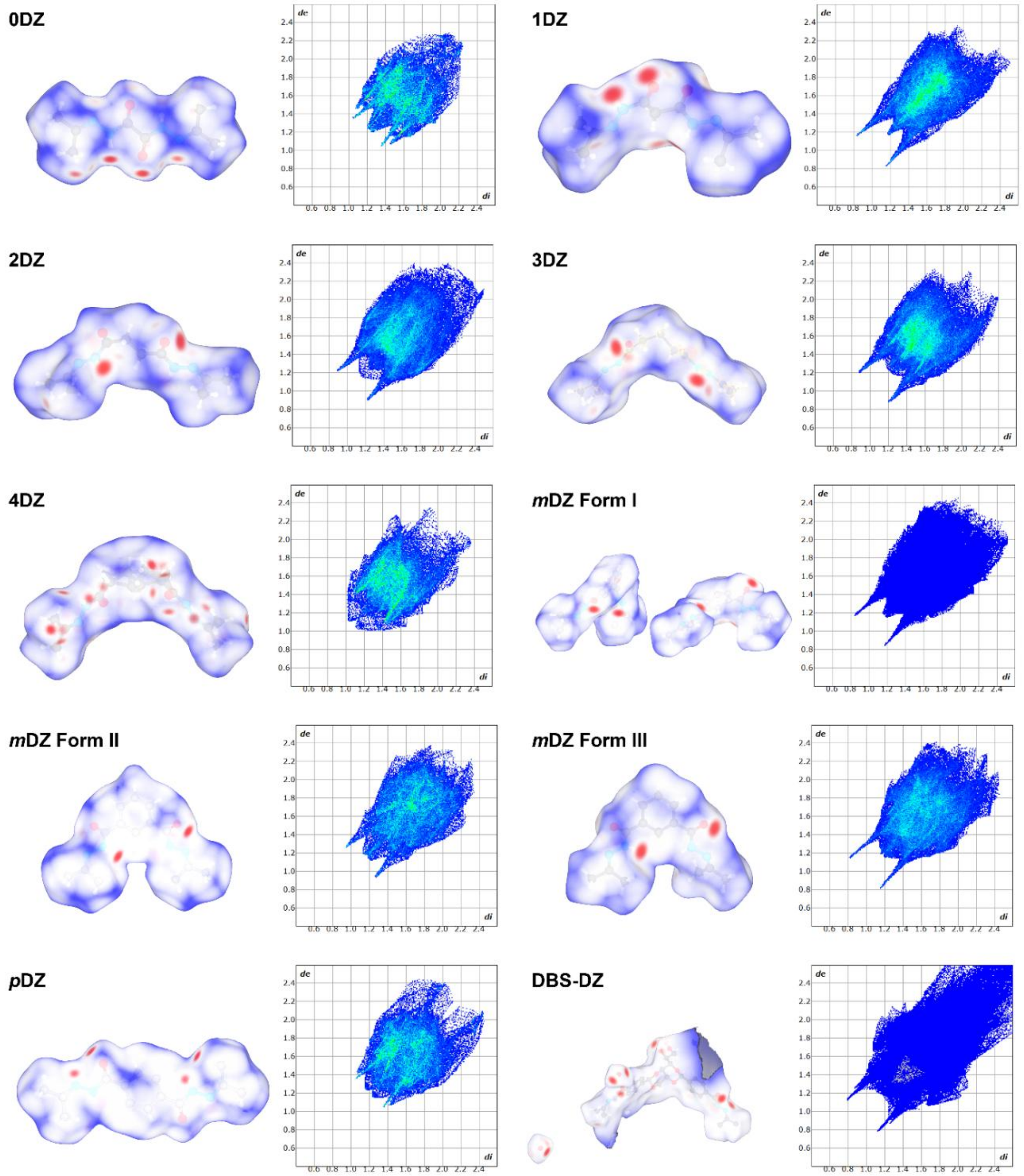

Figure S1. Calculated Hirshfeld surfaces (left) and corresponding 2D fingerprint plots (right) for each DZ structure.

Experimental Details

X-ray single-crystal diffraction data for *m*DZ Form II (repeat experiment), *m*DZ Form III, and DBS-DZ were collected at the I-19 synchrotron beamline (λ = 0.68890 Å) with a Dectris Pilatus 2M pixel-array photon-counting detector, undulator and graphite monochromator, at the Diamond Light Source, Oxfordshire. The data were processed using Xia2/DIALS,[5, 6] and *m*DZ Form III data were integrated using CrysAlisPro.[7] X-ray single-crystal diffraction data for the remaining structures were collected using Mo Kα radiation (λ = 0.71073 Å) on a Bruker D8Venture with a Photon III MM C14 CPAD detector, IμS-III-microsource, focusing mirrors diffractometer equipped with a Cryostream (Oxford Cryosystems 700+) open-flow nitrogen cryostat. All structures were solved using Olex2[8] with the ShelXT[9] structure solution program using Intrinsic Phasing and refined with the ShelXL[10] or Olex2.refine[11] refinement packages using Least Squares minimization on F^2. All non-hydrogen atoms were refined with anisotropic displacement parameters. Hydrogen atoms were located on the difference map after all other atoms had been refined. All other hydrogens were placed geometrically and refined using a riding model unless otherwise specified. For DBS-DZ the water within the large channels could not be sensibly modelled, therefore the electron density associated with the disordered water was removed from the refinement and accounted for using the PLATON SQUEEZE routine.[12]

Crystallographic data for all structures have been deposited with the Cambridge Crystallographic Data Centre (CCDC) with CCDC deposition numbers 2500873-2500883 and 2503351.

0DZ: Oxalyl dihydrazone was synthesized by the fast cooling of a near-boiling solution of 20 mg of oxalyl dihydrazide in 0.2 mL water:acetone (1:1), which resulted in clear colourless plate crystals as the solution reached room temperature. Crystal data: $C_8H_{14}N_4O_2$, M = 198.23 g/mol, 0.44 × 0.225 × 0.014 mm^3, monoclinic, space group $P2_1/n$ (no. 14), a = 5.1745(5) Å, b = 16.8291(15) Å, c = 5.6742(5) Å, β = 95.851(4) °, V = 491.55(8) Å^3, Z = 2, D_c = 1.339 g/cm^3, μ = 0.099 mm^{-1}, F(000) = 212.0, Mo Kα radiation, λ = 0.71073 Å, T = 120.0 K, $2\theta_{max}$ = 57.552 °, 13615 reflections collected. Final GooF = 1.239, R_1 = 0.0698 (1275 reflections with I > = 2σ(I)), wR_2 = 0.1255 (all data), 70 parameters, 0 restraints.

1DZ: Malonic dihydrazone was synthesized by refluxing 100 mg of malonic dihydrazide in 20 mL of acetone for two hours. 10 mg of the product was recrystallised from 0.05 mL of DMSO by slow cooling to give clear colourless needle crystals. Crystal data: $C_9H_{16}N_4O_2$, M = 212.26 g/mol, 0.261 × 0.066 × 0.04 mm^3, monoclinic, space group $P2_1/c$ (no. 14), a = 8.6380(4) Å, b = 16.7078(7) Å, c = 8.0144(3) Å, β = 103.0230(10) °, V = 1126.90(8) $Å^3$, Z = 4, D_c = 1.251 g/cm^3, μ = 0.091 mm^{-1}, $F(000)$ = 456.0, Mo Kα radiation, λ = 0.71073 Å, T = 120.0 K, $2\theta_{max}$ = 55.044 °, 28900 reflections collected. Final GooF = 1.138, R_1 = 0.0532 (2601 reflections with $I >= 2\sigma(I)$), wR_2 = 0.1132 (all data), 148 parameters, 0 restraints.

2DZ: Succinic dihydrazone was synthesized by refluxing 100 mg of succinic dihydrazide in 20 mL of acetone for two hours. The product was recrystallised by the antisolvent addition method: 5 mg of product was dissolved in 0.1 mL methanol and 0.1 mL of hexane was added to the crystallization vial to give clear colourless needle crystals. Crystal data: $C_{10}H_{18}N_4O_2$, M = 226.28 g/mol, 0.245 × 0.119 × 0.041 mm^3, monoclinic, space group $P2_1/n$ (no. 14), a = 11.8912(7) Å, b = 8.0793(5) Å, c = 13.1797(7) Å, β = 107.867(2) °, V = 1205.14(12) $Å^3$, Z = 4, D_c = 1.247 g/cm^3, μ = 0.089 mm^{-1}, $F(000)$ = 488.0, Mo Kα radiation, λ = 0.71073 Å, T = 120.0 K, $2\theta_{max}$ = 54.968 °, 29576 reflections collected. Final GooF = 1.090, R_1 = 0.0601 (2763 reflections with $I >= 2\sigma(I)$), wR_2 = 0.1463 (all data), 157 parameters, 0 restraints.

2DZ.4H_2O: Succinic dihydrazone tetrahydrate was synthesized by the slow evaporation of 0.4 mL acetone:water (1:1) in a solution with 40 mg succinic dihydrazide to give clear colourless plate crystals. Crystal data: $C_{10}H_{26}N_4O_6$, M = 298.34 g/mol, 0.252 × 0.197 × 0.018 mm^3, monoclinic, space group $P2_1/c$ (no. 14), a = 5.8990(2) Å, b = 6.1589(2) Å, c = 20.7730(6) Å, β = 92.626(1) °, V = 753.92(4) $Å^3$, Z = 2, D_c = 1.314 g/cm^3, μ = 0.107 mm^{-1}, $F(000)$ = 324.3, Mo Kα radiation, λ = 0.71073 Å, T = 120.0 K, $2\theta_{max}$ = 60 °, 28679 reflections collected. Final GooF = 1.057, R_1 = 0.0402 (2203 reflections with $I >= 2\sigma(I)$), wR_2 = 0.1041 (all data), 103 parameters, 0 restraints.

3DZ: Glutaric dihydrazone was synthesized by the slow cooling of 6 mg of glutaric dihydrazide in a 1:1 mixture of 0.2 mL DMSO:acetone to give clear colourless needle crystals. Crystal data: $C_{11}H_{20}N_4O_2$, M = 240.31 g/mol, 0.594 × 0.089 × 0.02 mm^3, monoclinic, space group $P2_1/n$ (no. 14), a = 9.0318(5) Å, b = 11.2712(6) Å, c = 12.3742(7) Å, β = 91.673(2) °, V = 1259.15(12) $Å^3$, Z = 4, D_c = 1.268 g/cm^3, μ = 0.090 mm^{-1}, $F(000)$ = 520.0, Mo Kα radiation, λ = 0.71073 Å, T = 120.0

K, $2\theta_{max}$ = 52.738 °, 21273 reflections collected. Final GooF = 1.097, R_1 = 0.0541 (2572 reflections with $I >= 2\sigma(I)$), wR_2 = 0.1244 (all data), 166 parameters, 0 restraints.

4DZ: Adipic dihydrazone was synthesized by the fast cooling of a near-boiling solution of 40 mg of adipic dihydrazide in 0.05 mL water:acetone (1:1), which resulted in clear colourless needle crystals as the solution reached room temperature. Crystal data: $C_{12}H_{22}N_4O_2$, M = 254.33 g/mol, 0.208 × 0.057× 0.026 mm^3, orthorhombic, space group *Fdd*2 (no. 43), a = 15.5691(9) Å, b = 28.4277(16) Å, c = 6.1100(4) Å, β = 90 °, V = 2704.2(3) Å^3, Z = 8, D_c = 1.249 g/cm^3, μ = 0.087 mm^{-1}, $F(000)$ = 1104.7, Mo Kα radiation, λ = 0.71073 Å, T = 120.0 K, $2\theta_{max}$ = 59.16 °, 23347 reflections collected. Final GooF = 0.983, R_1 = 0.0575 (1902 reflections with $I >= 2\sigma(I)$), wR_2 = 0.1259 (all data), 106 parameters, 212 restraints.

*o*DZ: Numerous avenues were employed in the pursuit of *o*DZ. Experiments included refluxing 1,2-benzenedicarboxylic acid dihydrazide (CAS 3645-45-2) in acetone and water:acetone (1:1), liquid assisted grinding of 1,2-benzenedicarboxylic acid dihydrazide with acetone, and slow cooling crystallization of 1,2-benzenedicarboxylic acid dihydrazide in acetone, water:acetone, DMSO:acetone, however, only IJOKIB could be crystallized. No impurities of IJOKIB were observed in the powder X-ray pattern of 1,2-benzenedicarboxylic acid dihydrazide. The formation of IJOKIB crystals and crystalline material occurred in solution and the solid state, with and without heating.

*m*DZ, Form I: Isophthalic dihydrazone, Form I was synthesized by the fast cooling of a near-boiling solution of 40 mg isophthalic dihydrazide in 0.3 mL of water:acetone (1:1) which resulted in clear colourless square plate crystals as the solution reached room temperature. Crystal data: $C_{14}H_{18}N_4O_2$, M = 274.32 g/mol, 0.171 × 0.161 × 0.026 mm^3, triclinic, space group *P*-1 (no. 2), a = 8.2836(4) Å, b = 8.3857(4) Å, c = 20.6240(10) Å, α = 84.501(2) °, β = 79.725(2) °, γ = 89.734(2) °, V = 1403.05(12) Å^3, Z = 4, D_c = 1.299 g/cm^3, μ = 0.090 mm^{-1}, $F(000)$ = 584.0, Mo Kα radiation, λ = 0.71073 Å, T = 120.0 K, $2\theta_{max}$ = 56.62 °, 42806 reflections collected. Final GooF = 1.056, R_1 = 0.0442 (6944 reflections with $I >= 2\sigma(I)$), wR_2 = 0.1004 (all data), 385 parameters, 0 restraints.

*m*DZ, Form II: Isophthalic dihydrazone, Form II was synthesized by the slow cooling of 160 mg isophthalic dihydrazide in a 5 mL water:acetone (1:1) mixture. 5 mg of the product was then filtered and recrystallised in 0.2 mL methanol to give clear colourless needle crystals, which were

subsequently placed in a desiccator to prevent any polymorph conversion. Crystal data: $C_{14}H_{18}N_4O_2$, M = 274.32 g/mol, 0.518 × 0.06 × 0.021 mm^3, monoclinic, space group $C2/c$ (no. 15), a = 19.475(2) Å, b = 8.9815(9) Å, c = 8.2696(8) Å, β = 100.066(3) °, V = 1424.2(2) Å^3, Z = 4, D_c = 1.279 g/cm^3, μ = 0.089 mm^{-1}, F(000) = 584.0, Mo Kα radiation, λ = 0.71073 Å, T = 200.0 K, $2\theta_{max}$ = 50.692 °, 14796 reflections collected. Final GooF = 1.094, R_1 = 0.0680 (1301 reflections with I > = 2σ(I)), wR_2 = 0.1399 (all data), 98 parameters, 0 restraints. *m*DZ Form II crystals were also collected the Diamond Light Source on different crystals to the original collection, but at the same temperature, and reveal the same polymorph. Crystal data: $C_7H_9N_2O$, M = 137.16 g/mol, 0.11 × 0.01 × 0.005 mm^3, monoclinic, space group $C2/c$ (no. 15), a = 19.529(3) Å, b = 8.9456(9) Å, c = 8.3152(9) Å, β = 100.602(11) °, V = 1427.9(3) Å^3, Z = 8, D_c = 1.276 g/cm^3, μ = 0.083 mm^{-1}, F(000) = 584.3, synchrotron radiation, λ = 0.68890 Å, T = 200.0 K, $2\theta_{max}$ = 48.82 °, 11856 reflections collected. Final GooF = 0.535, R_1 = 0.0571 (1264 reflections with I > = 2σ(I)), wR_2 = 0.1357 (all data), 112 parameters, 75 restraints.

*m*DZ, Form III: Isophthalic dihydrazone, Form III was synthesized by the slow cooling of 40 mg isophthalic dihydrazide in a 0.6 mL water:acetone (1:1) mixture. The clear, colourless needles were then filtered immediately and suspended in 0.1 mL ethyl acetate to slow the conversion of the needles to the plate morphology. Crystal data: $C_{14}H_{18}N_4O_2$, M = 274.32 g/mol, 0.115 × 0.006 × 0.005 mm^3, monoclinic, space group $P2_1/c$ (no. 14), a = 8.4173(5) Å, b = 8.1862(5) Å, c = 20.0715(17) Å, β = 95.615(7) °, V = 1376.40(17) Å^3, Z = 4, D_c = 1.324 g/cm^3, μ = 0.086 mm^{-1}, F(000) = 584.3, synchrotron radiation, λ = 0.68890 Å, T = 100.0 K, $2\theta_{max}$ = 47.98 °, 18945 reflections collected. Final GooF = 1.055, R_1 = 0.1167 (18945 reflections with I > = 2σ(I)), wR_2 = 0.3919 (all data), 193 parameters, 0 restraints. *m*DZ Form III crystals were also collected the Diamond Light Source in a slow cooling experiment from 200-100K on different *m*DZ needle crystals. The unit cell parameters match the previously collected 100 K data, but the data is of poor quality and therefore not included.

*p*DZ: Terephthalic dihydrazone was synthesized by the slow cooling of 12 mg of terephthalic dihydrazide in a 1 mL mixture of water:acetone (1:1) to give clear colourless plank crystals. Crystal data: $C_{14}H_{18}N_4O_2$, M = 274.32 g/mol, 0.343 × 0.16 × 0.04 mm^3, monoclinic, space group $P2_1/c$ (no. 14), a = 11.3237(18) Å, b = 7.8854(13) Å, c = 7.9632(11) Å, β = 107.616(5) °, V = 667.71(18) Å^3, Z = 2, D_c = 1.344 g/cm^3, μ = 0.093 mm^{-1}, F(000) = 292.0, Mo Kα radiation, λ = 0.71073 Å, T

= 120.0 K, $2\theta_{max}$ = 58.248 °, 17255 reflections collected. Final GooF = 1.032, R_1 = 0.0430 (1821 reflections with $I >= 2\sigma(I)$), wR_2 = 0.1171 (all data), 97 parameters, 0 restraints.

DBS-DZ was synthesized by the slow evaporation of acetone in a solution containing 1,3(*R*):2,4(*S*)-dibenzylidene-D-sorbitol bis(acylhydrazide), which yielded clear colourless needle crystals. Crystal data: $C_{28}H_{48}N_4O_{15}$, M = 680.711 g/mol, 0.104 × 0.013 × 0.011 mm^3, tetragonal, space group $P4_3$ (no. 78), a = 19.85982(19) Å, b = 19.85982(19) Å, c = 9.31354(17) Å, β = 90 °, V = 3673.37(7) Å^3, Z = 4, D_c = 1.231 g/cm^3, μ = 0.094 mm^{-1}, $F(000)$ = 1457.0, Synchrotron radiation, λ = 0.68890 Å, T = 100.0 K, $2\theta_{max}$ = 49.98 °, 62774 reflections collected. Final GooF = 1.007, R_1 = 0.0486 (7099 reflections with $I >= 2\sigma(I)$), wR_2 = 0.1268 (all data), 399 parameters, 1 restraint.

DBS-DZ solubility and gel screen: 30 solvents (2-picoline, diisopropyl ether, benzyl alcohol, chloroform, nitromethane, tetrahydrofuran, ethanol, methanol, mesitylene, acetonitrile, 1-propanol, 2-butanol, ethyl acetate, dichloromethane, dioxane, toluene, pyridine, cyclohexane, dimethylformamide, 1,3-dichlorobenzene, cyclopentanone, chlorobenzene, *p*-xylene, dimethyl sulfoxide (DMSO), nitrobenzene, water, acetone, dimethylacetamide (DMA), diethyl ether, and 2-butanone) at a gelator concentration of 0.5% wt/vol were explored. DBS-DZ is soluble in DMSO without heat, soluble in DMA with heat, and partially soluble in water with heat. DBS-DZ was insoluble in all other solvents attempted at 0.5 % wt/vol. At this concentration, the only solvent that yields a gel is water, as confirmed qualitatively by the vial inversion test.

References

1. A. Gavezzotti, Are Crystal Structures Predictable?, *Acc. Chem. Res.*, 1994, **27**, 309-314.
2. A. Gavezzotti and G. Filippini, Geometry of the intermolecular X-H...Y (X, Y=N, O) hydrogen-bond and the calibration of empirical hydrogen-bond potentials, *J. Phys. Chem.*, 1994, **98**, 4831-4837.
3. C. Macrae, I. Sovago, S. Cottrell, P. Galek, P. McCabe, E. Pidcock, M. Platings, G. Shields, J. Stevens, M. Towler and P. Wood, Mercury 4.0: from visualization to analysis, design and prediction, *J. Appl. Crystallogr.*, 2020, **53**, 226-235.
4. P. Spackman, M. Turner, J. McKinnon, S. Wolff, D. Grimwood, D. Jayatilaka and M. Spackman, CrystalExplorer: a program for Hirshfeld surface analysis, visualization and quantitative analysis of molecular crystals, *J. Appl. Crystallogr.*, 2021, **54**, 1006-1011.

5. G. Winter, xia2: an expert system for macromolecular crystallography data reduction, *J. Appl. Cryst.*, 2010, **43**, 186-190.
6. G. Winter, D. Waterman, J. Parkhurst, A. Brewster, R. Gildea, M. Gerstel, L. Fuentes-Montero, M. Vollmar, T. Michels-Clark, I. Young, N. Sauter and G. Evans, DIALS: implementation and evaluation of a new integration package, *Acta Crystallogr., D*, 2018, **74**, 85-97.
7. CrysAlisPro 1.171.44.85, Rigaku Oxford Diffraction/Agilent Technologies UK Ltd., 2024.
8. O. V. Dolomanov, L. J. Bourhis, R. J. Gildea, J. A. K. Howard and H. Puschmann, OLEX2: a complete structure solution, refinement and analysis program, *J. Appl. Crystallogr.*, 2009, **42**, 339-341.
9. G. Sheldrick, SHELXT - Integrated space-group and crystal-structure determination, *Acta Cryst. A*, 2015, **71**, 3-8.
10. G. M. Sheldrick, Crystal structure refinement with SHELXL, *Acta Cryst. C*, 2015, **71**, 3-8.
11. L. Bourhis, O. Dolomanov, R. Gildea, J. Howard and H. Puschmann, The anatomy of a comprehensive constrained, restrained refinement program for the modern computing environment-Olex2 dissected, *Acta Crystallogr., Sect. A:Found. Adv.*, 2015, **71**, 59-75.
12. A. Spek, PLATON SQUEEZE: a tool for the calculation of the disordered solvent contribution to the calculated structure factors, *Acta Crystallogr., Sect. C:Struct. Chem.*, 2015, **71**, 9-18.
